# A case report of a sigmoid perforation within an inguinoscrotal hernia sac caused by ingestion of a chicken wishbone

**DOI:** 10.1016/j.ijscr.2025.111062

**Published:** 2025-02-16

**Authors:** E.L. Claassens, M.M.G. van Berckel, K.P. Wevers

**Affiliations:** a Department of Surgery, Maastricht University Medical Center, P. Debyelaan 25, 6229 HX Maastricht, the Netherlands; b Department of Surgery, Catharina Hospital, Michelangelolaan 2, 5623 EJ Eindhoven, the Netherlands

**Keywords:** Case report, Foreign body, Chicken wishbone, Sigmoid perforation, Inguinoscrotal hernia, Lichtenstein surgery

## Abstract

**Introduction and importance:**

Ingestion of foreign bodies is common and often harmless. However sharp objects like bones can cause intestinal perforations, particularly at anatomical narrowing points. Diagnosis is challenging due to patients' lack of recollection of ingestion and subtle imaging findings. We present a rare case of a sigmoid perforation within an inguinoscrotal hernia sac caused by a chicken wishbone, discovered during elective hernia repair surgery.

**Case presentation:**

A 66-year-old male presented with a left inguinoscrotal hernia causing mild discomfort, but without signs of local infection or an acute abdomen. Preoperative imaging showed sigmoid colon within the hernia sac without complications. During elective surgery, pus and fecal material were drained from the hernia sac, revealing a perforated sigmoid colon with a chicken wishbone protruding. Eight centimeters of inflamed sigmoid colon were resected, and primary anastomosis was performed given the contained perforation and the patient's stable condition. Postoperative complications included anastomotic dehiscence, necessitating further surgeries, however the patient recovered well and remains in good health at 12-month follow-up.

**Clinical discussion:**

This case highlights the diagnostic challenge of intestinal perforation by foreign bodies, especially without acute symptoms. Omental adhesion likely sealed the perforation, preventing peritonitis.

**Conclusion:**

A sigmoid perforation within an inguinoscrotal hernia sac caused by a chicken wishbone is rare. Effective management requires careful postoperative care. This case contributes to the understanding of intestinal perforations caused by foreign bodies and their unique challenges.

## Highlights

• Ingested foreign bodies often traverse the GI tract without issues
• Sharp objects can cause perforations, especially at angulation points
• Case involves a chicken wishbone perforating the sigmoid within a hernia sac
• Highlights diagnostic and surgical challenges in rare intestinal perforation cases

## 1 Introduction

Ingestion of foreign bodies is a relatively common occurrence. In the majority of these cases, the foreign body traverses the gastrointestinal tract without any adverse outcomes. However, in some cases, intestinal perforation can occur due to ingestion of sharp objects such as bones or toothpicks, most commonly at points of angulation or narrowing such as the terminal ileum or rectosigmoid junction [[1], [2], [3], [4]]. Diagnosis of intestinal perforation caused by foreign bodies remains difficult, since the foreign bodies are often subtle and therefore not or minimally visible on imaging. Additionally, most patients have no recollection of ingesting a foreign body [5]. In general, patients with intestinal perforation present with acute abdomen, which consists of abdominal pain, fever, peritonitis and/or intra-abdominal abscesses or fistulas [6]. We report a case of a patient without signs of an acute abdomen with a perforated sigmoid colon within an inguinoscrotal hernia sac caused by a chicken wishbone, found during an elective hernia repair surgery. We aim to present this unusual presentation and the challenges we encountered while treating this patient.

## 2 Case presentation

**Fig. 1 + 2:**
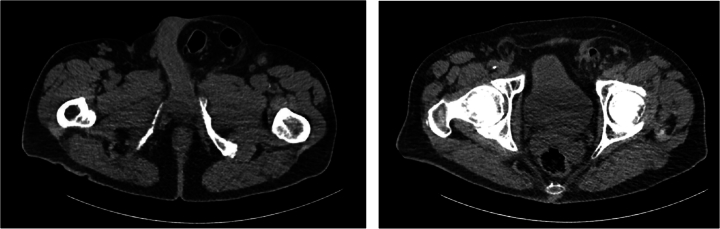
Left sided inguinal hernia on CT-abdomen eight months prior to presentation on the outpatient surgery clinic.

**Fig. 3:**
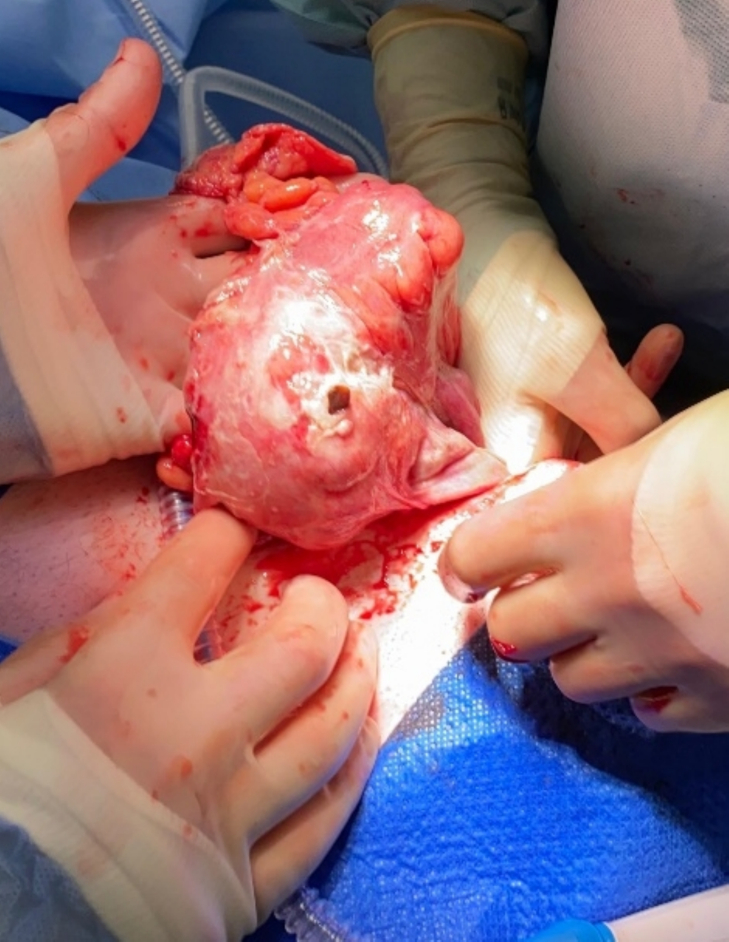
Perforated sigmoid colon with chicken wishbone sticking out.

A 66-years-old male presented to the outpatient clinic with swelling in the left inguinoscrotal region, present since multiple years with since more recently occasional abdominal pain during the night. His medical history included hypertension and hypercholesterolemia for which he used carbasalate calcium in the context of cardiovascular risk management, and a right sided inguinal hernia for which he underwent a Lichtenstein repair in 2009. Additionally, he had a six pack-year history of smoking. Clinical examination showed a left sided, partly reducible scrotal hernia. Preoperative ultrasound of the left inguinoscrotal region revealed an inguinal hernia with bowel present inside the hernia sac without signs of complications. On an abdominal CT from eight months prior, requested by the urologist due to suspicion of urolithiasis, a left sided inguinal hernia containing sigmoid colon was seen (Fig. 1 + 2). Because of mild discomfort the patient reported from the hernia he was scheduled for elective hernia repair surgery. The surgery took place 23 days after the initial consultation during which the patient did not indicate any change of complaints. Preoperatively, the surgical team noticed a hard non-reducible mass in the scrotum which was slightly warm and minimally red. A Lichtenstein approach was chosen. The procedure was performed by an experienced gastrointestinal surgeon, with extensive experience in inguinal hernia repairs (>100 per year). During surgery, when the hernia sac was opened, pus and fecal material drained from the scrotal part of the hernia sac. The sigmoid was reduced from the scrotum and a perforation of the sigmoid colon with a chicken wishbone sticking out was exposed (Figs. 3 + 4). The omentum was wrapped around the perforated part of the sigmoid. Inspection of the sigmoid colon revealed eight centimeters of affected sigmoid due to inflammation and was therefore resected. Because of the contained aspect of the perforation and the recently unaltered good and non-septic condition of the patient, restoration of continuity by an end-to-end anastomosis was chosen. The anastomosis was performed through the groin incision, subsequently the abdominal wall was primarily closed and a penrose drain was placed in the surgical site. The patient was admitted to the surgical ward and postoperative intravenous antibiotics were started. One day postoperatively the patient had a decrease in Hemoglobin level for which two packed cells were administered. There were no clinical signs of an ongoing bleeding and during the subsequent postoperative course, the hemoglobin level remained stable. On postoperative day four, the patient had a subfebrile temperature and an increase in infection parameters (C-reactive protein 244 mg/L, leucocytes 20.2 10*9/L) but clinical performance remained good and he was able to walk around the ward nevertheless. An abdominal CT showed a large intra-abdominal hematoma and a limited amount of free air and fluid, possibly indicating an anastomotic leak (Fig. 5). A diagnostic laparoscopy was performed five days postoperatively to evacuate the large intra-abdominal hematoma. There was no fecal peritonitis and the omentum had covered the anastomosis*.* Initially, the patient improved clinically however, a chest X-ray was taken on postoperative day seven as there was a decrease in oxygen saturation to 96 % with 2 L oxygen via nasal catheter, which showed excessive subdiaphragmatic free air. An explorative laparoscopy was performed with conversion to laparotomy due to anastomosis dehiscence for which an end colostomy was formed. Postoperatively, the patient was admitted to the ICU due to vasopressor requirement but he improved rapidly and was able to return to the surgical ward after 24 h. The patient developed a postoperative ileus for which he received parenteral feeding during three days, after which the ileus resolved. The patient was discharged in good clinical condition on postoperative day fifteen after the initial surgery. Histopathological examination of the resected part of the sigmoid did not reveal additional findings. Follow-up was done in the outpatient department, the patient is currently twelve months after the initial surgery and doing well. This manuscript has been conducted in alignment with the SCARE criteria [7].

**Fig. 4:**
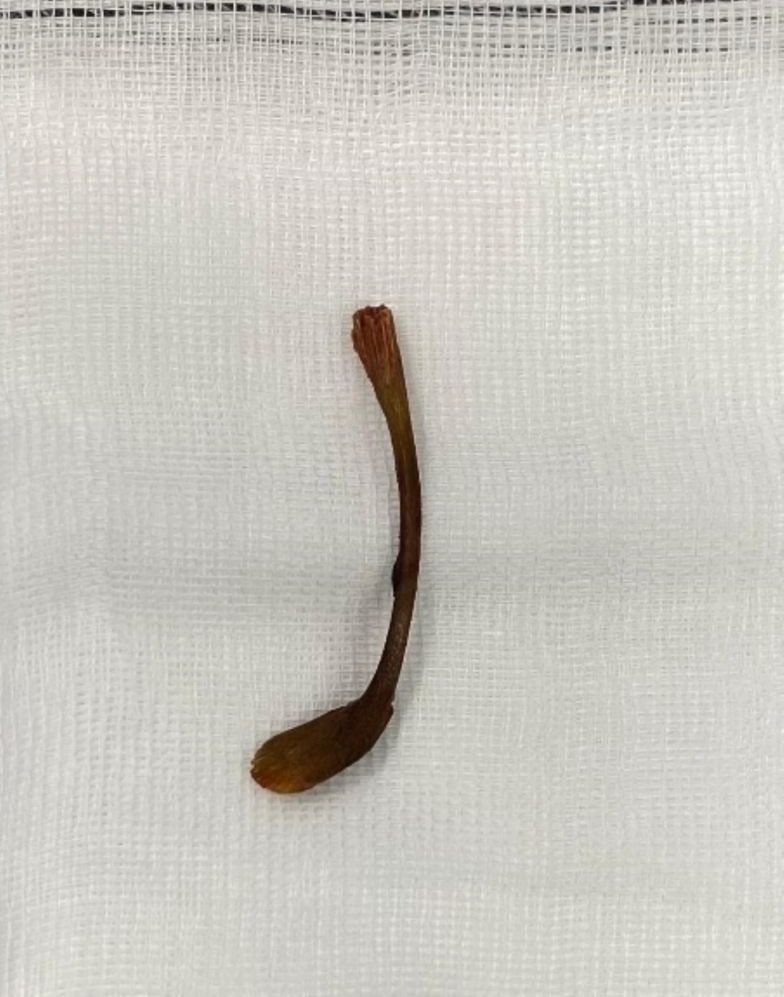
Chicken wishbone, found during surgery.

**Fig. 5:**
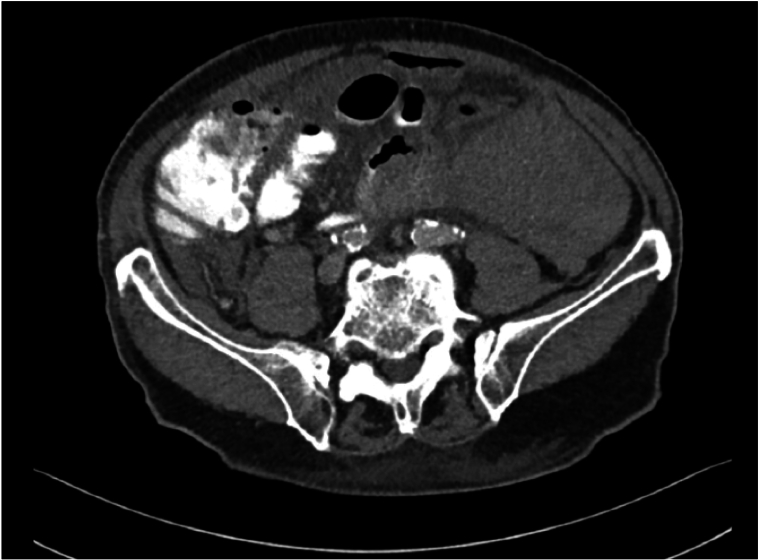
POD 4, large intra-abdominal hematoma left abdomen and limited amount of free air and fluid.

## 3 Discussion

This case highlights a rare presentation of a sigmoid perforation within an inguinoscrotal hernia sac caused by the ingestion of a chicken wishbone. Intestinal perforations due to foreign bodies are quite uncommon, and their diagnosis is often challenging due to the nonspecific symptoms and the absence of evidence for ingestion in the case history. In this case, the absence of acute abdominal symptoms and the atypical presentation within a hernia sac further complicated the diagnostic process. Preoperative imaging did not reveal signs of complications and the patient's mild symptoms did not raise immediate concerns for an acute abdomen, underscoring the diagnostic difficulty.

In literature, bowel perforation due to the ingestion of a foreign body is quite frequently described. However, a perforation occurring inside a hernia sac that presents during elective surgery caused by a chicken bone is very rare. Some cases of perforations in hernia sacs due to a foreign body have been described [8,9]. The most remarkable aspect is that our patient did not show any signs of an acute abdomen or systemic signs of a local infection, indicated by fever, tachycardia and/or abdominal pain. A possible explanation is that the omentum adhered to the sigmoid perforation, effectively sealing it and thereby preventing the occurrence of fecal peritonitis. The omentum is known to wrap around areas of injury or infection. Logically, the omentum can wrap around perforations caused by foreign bodies as well, giving the injury the possibility to heal gradually, as mentioned in a recent case report [10]. Moreover, preoperative imaging nine months prior to surgery showed no signs of a foreign body being present within the inguinal hernia sac, even upon retrospective review, indicating that the wishbone was ingested after imaging. It is possible that at the time of the operation, the perforation caused by the ingested wishbone had not been present for long enough for the patient to become ill since there were no signs of peritonitis before and during surgery. Remarkably, recently a case report was published that reports about a sigmoid colon perforation due to a chicken wishbone, but intra-abdominally [2]. Initially, their patient reported with recurrent constipation. An abdominal CT scan revealed a thickened sigmoid colon and an intraluminal hyperdensity. Conservative management was attempted however, after a few weeks the patient presented with worsening abdominal pain and hydronefrosis on CT scan caused by a pelvic collection. Emergency laparotomy revealed a sigmoid inflammatory mass and a chicken wishbone in the pelvis. Sigmoid resection and primary anastomosis were performed. In our case, the decision to perform a resection of the affected sigmoid segment and create an end-to-end anastomosis was based on the contained nature of the perforation and the patient's stable, non-septic condition at the time of surgery. This decision aligns with the surgical principles of addressing the source of contamination while attempting to restore bowel continuity when feasible. The surgery was complicated by anastomosis dehiscence for which the patient underwent two additional surgeries, which highlights the inherent risks associated with primary anastomosis in contaminated fields and the challenges in predicting postoperative outcomes.

Remarkably, chicken wishbones specifically are frequently mentioned in case reports concerning the ingestion of foreign bodies [2,11]. Several reasons possibly contribute to the relatively frequent ingestion of chicken bones. The wishbone is small and therefore easily overlooked, making it more likely to be accidentally swallowed. Additionally, chicken is often prepared in ways that leave the bones more accessible, such as roasting, where bones may be present while the food is served. Moreover, chicken is a common and popular food worldwide, leading to higher overall consumption rates. This naturally increases the chances of ingestion of chicken bones.

## 4 Conclusion

The management of sigmoid perforation within an inguinoscrotal hernia sac caused by a chicken wishbone requires careful surgical planning and diligent postoperative care. While primary anastomosis may be attempted in stable patients, the risk of complications necessitates close monitoring and a readiness to intervene surgically if needed. By sharing our experience, we hope to contribute to the existing literature on intestinal perforations caused by foreign bodies.

## CRediT authorship contribution statement

We collaboratively contributed to this case report.

EL Claassens: collection and analysing of the clinical data, obtaining informed consent, literature review, drafting of the manuscript

MMG van Berckel: analysing of clinical data, reviewing manuscript and critical revisions

KP Wevers: analysing of clinical data, reviewing manuscript and critical revisions, supervision

All authors reviewed and approved the final version of the manuscript for submission

## Informed consent

Written informed consent was obtained from the patient for publication of this case report and accompanying images. A copy of the written consent is available for review by the Editor-in-Chief of this journal on request.

## Ethical approval

This study was exempt from ethical approval since it involves a case report rather than a broader clinical study. However, written informed consent was obtained from the patient for the publication of this case report and the included images. This consent ensures that the patient fully understands the implications of the publication and has voluntarily agreed to share their case details. The consent form is uploaded separately.

## Guarantor

Eva Claassens – PhD Candidate department of Surgery

Maastricht University

eva.claassens@maastrichtuniversity.nl

## Research registration number

Not applicable.

## Sources of funding

This research did not receive any specific grant from funding agencies in the public, commercial, or not-for-profit sectors.

## Declaration of competing interest

None.

## Data Availability

The authors confirm that the data supporting the findings of this study are available within the article.
